# Efficacy of Intra-articular Super-dose Platelet-Rich Plasma Injection in Improving Pain in a Middle-Aged Sedentary Female With Meniscal Tear: A Case Report

**DOI:** 10.7759/cureus.70059

**Published:** 2024-09-23

**Authors:** Sneha Thirugnana Sambandam, Dobson Dominic, Praveen Ravi, Ashirwad Jadhav

**Affiliations:** 1 Sports Medicine, Saveetha Medical College and Hospitals, Saveetha Institute of Medical and Technical Sciences, Chennai, IND

**Keywords:** growth factors, high volume prp, meniscus injury, meniscus rehabilitation, prp, regenerative medicine, regenerative medicine therapies

## Abstract

This case study explores the use of super-dose platelet-rich plasma (PRP) in managing a meniscal tear in a 43-year-old woman with left knee pain. The patient was diagnosed with a vertical longitudinal tear of the body and posterior horn medial meniscus, confirmed through magnetic resonance imaging. Super-dose PRP, characterized by a higher platelet volume (8 ml), was administered intra-articular. Post-injection, the patient reported initial discomfort for a few days, but significant improvements in pain and range of motion (ROM) were observed within the first week. Over 12 weeks, her visual analog scale pain score decreased from 8/10 during activity to 2/10, and her ROM returned to full functionality. The case supports the hypothesis that super-dose PRP may enhance the healing process in meniscal injuries by promoting tissue healing and regeneration through the release of growth factors, angiogenesis, collagen synthesis, and inflammation modulation. This case highlights PRP's potential as a non-surgical treatment for meniscal tears. Further large-scale studies and randomized controlled trials are needed to validate these results, optimize dosing strategies, and determine which patient populations will benefit most from this innovative treatment approach.

## Introduction

The meniscus is a fibro-cartilaginous structure that is an essential component of the knee joint and has functional, biomechanical, and anatomical significance [1]. The incidence of meniscal tears is estimated at 60 per 100,000 individuals, although this statistic is possibly a significant underestimation of the true rate [1]. According to the research, individuals with known meniscal injuries have faster cartilage degradation, leading to early osteoarthritic changes making meniscal tear management all the more crucial [1]. 

Previously, total meniscectomy was the gold standard for managing meniscal injuries until the 1970s. In recent decades, meniscus preservation surgeries have demonstrated a high success rate due to their faster recovery and enhanced functional outcomes [1,2]. However, conservative approaches utilizing exercise therapy as stand-alone or along with pharmacological interventions have proven beneficial in managing meniscal injuries [2,3]. Recent studies have demonstrated the role of regenerative medicine in enhancing the body’s natural healing process [4,5]. Among the various regenerative modalities, platelet-rich plasma (PRP) has gained significant attention due to its efficacy in treating musculoskeletal injuries. However, its effectiveness in the management of meniscal tears remains fully explored.

A super-dose PRP is characterized by a higher volume of platelets than standard PRP preparation [6]. The standard volume of PRP used is 4 ml, partly because most commercial equipment that synthesizes PRP produces only 3 to 5 ml of PRP. In their study, Dhillon et al. defined super-dose PRP as “8 ml” of PRP [6]. The rationale behind the usage of super-dose lies in the dose-dependent nature of its therapeutic effect. It is hypothesized that the higher volume of platelets in the PRP enhances the delivery of growth factors and cytokines to the injury site [3-5]. A benchmark study by Patel et al. demonstrated the positive effects of super-dose PRP in knee osteoarthritis [7]. This approach is particularly of interest in meniscal tears where the avascular nature of the inner thirds of the meniscus limits its healing capacity.

Though the efficacy of intraarticular PRP has been explored, there is limited evidence on the outcomes of super-dose PRP in managing meniscal tears. This case study aims to examine the use of super-dose PRP in the treatment of a knee meniscal body and posterior horn tear of a middle-aged woman. Through a detailed analysis of the post-injection reaction and functional outcomes of the patient, this report will provide valuable insights into the feasibility and efficacy of this novel therapeutic approach.

## Case presentation

A 43-year-old sedentary female presented to the Sports Medicine OPD of Saveetha Medical College and Hospitals, Chennai, with complaints of left knee pain for two months. She was a homemaker by profession and performed daily routine activities such as washing vessels, cooking, and mopping the floor. The pain was present in front of the knee, had started insidiously, and was gradually progressive, and she had a VAS (Visual Analog Scale) pain score of 6/10 during rest which increased to 8/10 during physical activity. The patient also gave a history of clicks while walking but no history of swelling, instability, or locking of the knee was reported. The patient had no history of similar complaints in the past and also had no co-morbidities. Her physical examination revealed no significant findings and her vitals were within normal limits. A local examination of the left knee showed a limited range of motion (ROM) of 0-120 degrees with painful terminal flexion. Further examination revealed positive tests for joint line tenderness, McMurray, and Thessaly tests, and she had a Trendelenburg gait.

**Figure 1:**
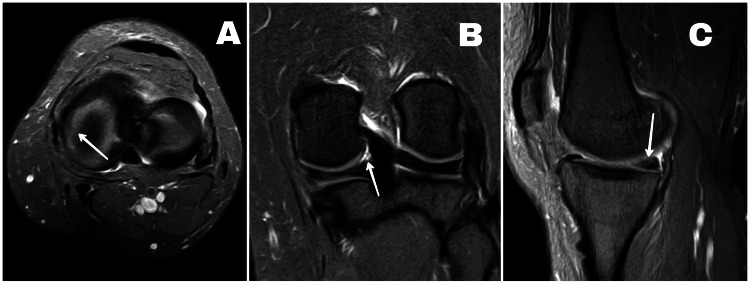
(A) Axial T2-weighted magnetic resonance image of left knee, (B) coronal T2-weighted magnetic resonance image of left knee, and (C) sagittal T2-weighted magnetic resonance image of left knee. The arrow in image A shows a vertical body tear of the medial meniscus, the arrow in image B shows a posterior horn tear of the medial meniscus, while the arrow in image C shows posterior horn tear of the medial meniscus.

Laboratory investigations were normal (C-reactive protein - 2 milligram/liter, erythrocyte sedimentation rate - 8 millimeters/hour, white blood cells - 5.4 x 109/liter, platelet count - 2,50,000/microliter). Plain anteroposterior and lateral radiographs of the left knee were advised which revealed no significant findings. Magnetic resonance imaging (MRI) was later advised which showed a vertical longitudinal tear involving the body and posterior horn of the medial meniscus with adjacent mild soft tissue edematous changes (Figures 1A, 1B, 1C). Early osteoarthritic changes of the medial tibio-femoral joint were also noted on the MRI with no other significant findings. The patient was advised admission for intra-articular injection of super-dose PRP injection.

**Figure 2:**
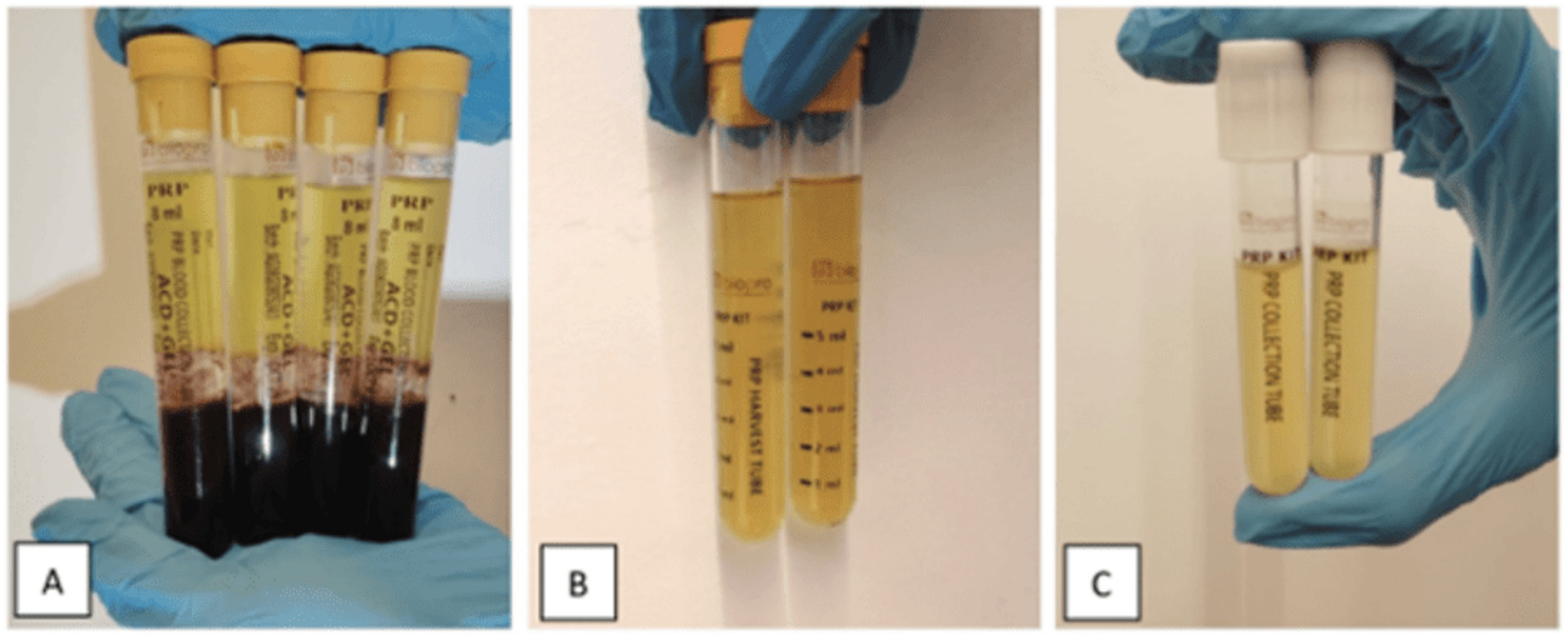
(A) PRP after the first spin, (B) PRP after the second spin, and (C) the final PRP in the collection tube (4 ml + 4 ml). PRP, platelet-rich plasma.

The PRP was prepared by collecting 28 ml of venous blood sample (in 4 tubes) from the patient. A BioPro PRP kit (Port Huron, Michigan, USA) was used to synthesize the PRP. The withdrawn blood was mixed with acid-citrate dextrose (ACD) and left to rest for five minutes. Post the resting period, the tubes were placed in a centrifuge machine (REMI, Mumbai, India) and a double spin method was used to obtain PRP (first spin at 2500 rpm for five minutes and second spin at 3800 rpm for 12 minutes) (Figures 2A, 2B). The PRP is then transferred to a collecting tube and pH regular is added (Figure 2C). Prior to the injection of PRP, calcium chloride (0.2 ml) which is a PRP activator was added to the sample making it a total of 8 mL of PRP with a platelet concentration of 10.24 lakh/microliter.

After obtaining informed consent, an intra-articular PRP injection (8 mL) was administered by an anterolateral approach in the left knee using a 23-gauge (1.5-inch) needle. The injection was administered with the patient in a supine position with hips flexed at 45 degrees and knees bent at 90 degrees. Povidine iodine (10%) was used to sterilize the injection site and the sports medicine physician wore sterile gloves while performing the procedure. No local anesthetic was used prior to the administration of PRP injection.

The patient reported pain during the procedure (VAS - 8/10) and immediately after the procedure (VAS - 9/10). Post-injection administration cryotherapy was administered for a period of 15 minutes post which VAS score decreased to 6/10. The patient was advised antibiotics (doxycycline 100 mg given twice daily for three days) as a preventative measure along with cryotherapy and rest for the next 24 hours. Four hours post the injection patient reported pain (VAS - 8/10) and stiffness (ROM - 0-20 degrees). The patient was reassured and cryotherapy was advised every second hourly. Sixteen hours post the injection, the pain had improved (VAS - 6/10) but stiffness persisted (ROM - 0-30 degrees). Forty-eight hours post the procedure pain and stiffness improved (VAS - 4/10, ROM - 0 - 70 degrees). The patient was discharged and asked to report to the OPD after a week. She was advised to do knee ROM exercises along with knee isometric exercises.

One week post the injection, the patient reported significant improvement in pain compared to pre-procedure levels (VAS-3/10) and ROM was restored (0 - 125 degrees). She was advised to engage in lower limb strengthening exercises. Two weeks post the injection, she was prescribed multiplanar movements for the lower limb along with proprioception exercises and the exercises were progressively increased in terms of complexity, intensity and volume over six weeks. Six weeks post the injection her VAS score was 2/10 and she was compliant with all her exercises. She continued her rehabilitation protocol for six more weeks. Her detailed 12-week rehabilitation protocol is given in Table 1. At the 12-week follow-up period, she reported significant pain improvement (no pain during any physical activity) and improved function.

**Table 1:** Twelve-week rehabilitation program.

Rehabilitation phase	Time post injection	Primary goal	Exercise	Criteria for progression
1	0 - 7 days	Control pain and swelling	Relative rest and activities as tolerated	Pain - 3/10 and absence of swelling
			Knee isometric exercises	
			Active and passive range of motion exercises	
2a	8 - 14 days	Improve range of motion	Knee isometric exercises	Pain - 2/10, restoration of range of motion
			Active and passive range of motion exercises	
			Strengthening exercises of lower limb and core (like squats, clams, lunges, leg raises, glute bridges, dynamic hip movements with a resistance band, calf raises, planks, v-sit, crunches, cat camel) done for 2-3 sets with 8-10 repetitions in each set	
2b	2 - 6 weeks	Develop strength	Aerobic exercises as tolerated (Walking, stationary cycling)	Pain - 2/10
			Exercises done at a higher volume than level 2a (3-4 sets of 10-15 repetitions each)	
			Balance and proprioception exercises (single-leg balance, tandem walking)	
3	6 - 12 weeks	Develop dynamic neuromuscular control	Plyometric exercises (double leg hops, star jumps, step-ups, step-downs, box jumps)	Pain - 2/10
			Strengthening exercises done at higher resistance and repetitions than level 2b (3-4 sets of 15-20 repetitions each)	
			Aerobic exercises (walking, stationary cycling) done at a higher duration than level 2b (20-30 minutes) along with increased duration of balance and proprioceptive training	

After completing the 12-week intervention, the patient expressed satisfaction with her chosen treatment option. She acknowledged experiencing some pain in the days immediately following the injection but indicated that she was willing to tolerate it and still preferred this treatment over surgery.

## Discussion

This case report details the use of a super-dose PRP injection in treating a middle-aged sedentary female with a meniscal tear. The patient experienced significant pain relief and functional improvement over a 12-week period following the intra-articular PRP injection. This outcome suggests that super-dose PRP could be a viable therapeutic option for managing meniscal tears, particularly in patients who may not be ideal candidates for surgery or who prefer a more conservative approach.

The use of PRP in musculoskeletal injuries, including meniscal tears, has gained considerable attention due to its potential to enhance the body's natural healing processes. PRP is rich in growth factors, cytokines, and other bioactive proteins that can stimulate tissue repair and reduce inflammation [3,4]. It enhances tissue repair and regeneration by releasing growth factors such as platelet-derived growth factor, transforming growth factor-beta, vascular endothelial growth factor, and epithelial growth factor [4,5]. These growth factors stimulate cell proliferation, angiogenesis, and collagen synthesis. It also modulates the inflammatory response, promoting faster healing and reducing tissue damage [3-5]. However, the effectiveness of PRP in meniscal injuries, particularly when used in a super-dose format, is still under investigation.

In this case, the patient reported a reduction in pain from a VAS score of 6/10 at rest and 8/10 during activity before the procedure to 2/10 at the 12-week follow-up. Additionally, the ROM improved from 0-120 degrees pre-injection to 0-125 degrees one week post-injection and was fully restored at the 12-week mark. These results are consistent with the hypothesis that a higher volume of platelets in super-dose PRP may enhance the delivery of growth factors to the injury site, thereby promoting healing in the relatively avascular meniscal tissue.

The findings of this case align with the outcomes reported in recent studies on the use of PRP in meniscal injuries. A systematic review by Patel et al. evaluated the efficacy of PRP in treating meniscal tears and reported that PRP injections led to significant improvements in pain and function in most patients, with outcomes comparable to those observed in this case [8]. The review highlighted that while standard PRP volumes were generally effective, the possibility of enhanced outcomes with higher doses like super-dose PRP warrants further investigation [8].

Dhillon et al. conducted a randomized controlled trial (RCT) comparing the effects of standard and super-dose PRP in patients with osteoarthritis [6]. The study found that patients receiving super-dose PRP had a more significant reduction in pain and faster functional recovery than those who received standard doses [6]. These findings support the notion that a dose-dependent effect exists, where a higher concentration of platelets may translate to better clinical outcomes, as observed in this case [6].

However, not all studies have reported uniformly positive outcomes. For example, a study by Li and Weng reported mixed results regarding the efficacy of PRP in meniscal injuries [9]. While some patients experienced significant pain relief and functional improvements, others showed minimal or no improvement [9]. The authors suggested that factors such as the severity of the tear, the specific location within the meniscus, and the patient's overall health might influence the efficacy of PRP [9]. In this case, the patient's relatively good health and the location and type of the tear might have contributed to the positive outcome [10].

Lastly, a study by Thu AC examined the role of PRP in combination with other therapies, such as physical therapy and exercise, and concluded that a multimodal approach might yield the best results [11]. In the present case, the patient’s structured rehabilitation program, which included ROM and strengthening exercises, likely played a crucial role in her recovery. This finding is consistent with the notion that PRP should not be viewed as a standalone treatment but as part of a comprehensive management plan.

The absence of a post-injection MRI is a limitation of this study, but it is justified by the significant clinical improvements observed, including reduced pain and restored function. Given these positive outcomes, further imaging was deemed unnecessary, as it would not have changed the management approach. Additionally, MRI is a resource-intensive procedure, and its use is typically reserved for cases where clinical outcomes are uncertain or further intervention is being considered.

## Conclusions

The present case study investigated the use of super-dose PRP for the treatment of a medial meniscal body and posterior horn tear in a 43-year-old female patient who experienced pain in her left knee. This study supports the potential efficacy of super-dose PRP (8 ml) in managing meniscal tears, particularly in patients who may not be suitable candidates for surgery or those seeking less invasive treatment options. PRP has a high concentration of growth factors, cytokines, and other bioactive proteins that have the ability to promote tissue regeneration and decrease inflammation. Further research, particularly large-scale randomized control trials, is needed to establish standardized protocols for super-dose PRP use in meniscal injuries and to identify the patient populations most likely to benefit from this treatment.
